# Impact of pre-dialysis nephrology care engagement and decision-making on provider and patient action toward permanent vascular access

**DOI:** 10.1186/s12882-021-02264-7

**Published:** 2021-02-16

**Authors:** Vanessa Grubbs, Bernard G. Jaar, Kerri L. Cavanaugh, Patti L. Ephraim, Jessica M. Ameling, Courtney Cook, Raquel C. Greer, L. Ebony Boulware

**Affiliations:** 1 Division of Nephrology, San Francisco/ San Francisco General Hospital Renal Center, University of California, Box 1341, 1001 Potrero Avenue, Bldg 100, Room 342, San Francisco, CA 94110 USA; 2 Department of Medicine, Johns Hopkins School of Medicine, Baltimore, MD USA; 3 Department of Epidemiology, Johns Hopkins Bloomberg School of Public Health, Baltimore, MD USA; 4 Welch Center for Prevention, Epidemiology and Clinical Research, Johns Hopkins University, Baltimore, MD USA; 5 Nephrology Center of Maryland, Baltimore, MD USA; 6 Division of Nephrology & Hypertension, Vanderbilt University Medical Center, Nashville, TN USA; 7 Department of Internal Medicine, University of Michigan Medical School, Ann Arbor, MI USA; 8 Patient Safety Enhancement Program, University of Michigan and VA Ann Arbor Healthcare System, Ann Arbor, MI USA; 9 Duke University Medical Center, Durham, NC UK

**Keywords:** Hemodialysis, Vascular access, Pre-dialysis nephrology care, Patient decision-making

## Abstract

**Background:**

While catheters are often thought the result of emergency hemodialysis (HD) initiation among patients with little or no pre-dialysis nephrology care, the role of patient level of engagement in care and modality decision-making have not been fully explored.

**Methods:**

This is a retrospective medical record review of adults (age 18–89 years) who received care in academically affiliated private practice, public hospital, or Veterans Administration settings prior to initiating HD with a catheter between 10/1/2011 and 9/30/2012. Primary predictors were level of patient engagement in nephrology care within 6 months of HD initiation and timing of modality decision-making. Primary outcomes were provider action (referral) and any patient action (evaluation by a vascular surgeon, vein mapping or vascular surgery) toward [arteriovenous fistula or graft, (AVF/AVG)] creation.

**Results:**

Among 92 incident HD patients, 66% (*n* = 61) initiated HD via catheter, of whom 34% (*n* = 21) had ideal engagement in care but 42% (*n* = 25) had no documented decision. Providers referred 48% (*n* = 29) of patients for AVF/AVG, of whom 72% (*n* = 21) took any action. Ideal engagement in care predicted provider action (adjusted OR 13.7 [95% CI 1.08, 175.1], *p* = 0.04), but no level of engagement in care predicted patient action (*p* > 0.3). Compared to patients with no documented decision, those with documented decisions within 3, 3–12, or more than 12 months before initiating dialysis were more likely to have provider action toward AVF/AVG (adjusted OR [95% CI]: 9.0 [1.4,55.6], *p* = 0.2, 37.6 [3.3423.4] *p* = 0.003, and 4.8 [0.8, 30.6], *p* = 0.1, respectively); and patient action (adjusted OR [95% CI]: 18.7 [2.3, 149.0], *p* = 0.006, 20.4 [2.6, 160.0], *p* = 0.004, and 6.2 [0.9, 44.0], *p* = 0.07, respectively).

**Conclusions:**

Timing of patient modality decision-making, but not level of engagement in pre-dialysis nephrology care, was predictive of patient and provider action toward AVF/AVG Interventions addressing patients’ psychological preparation for dialysis are needed.

## Background

Advance preparation for initiating renal replacement therapy (RRT) in patients with progressive chronic kidney disease (CKD), including appropriate dialysis access and outpatient start, represents a cornerstone of optimal therapy and is associated with significant health benefits. With few exceptions, permanent vascular access (arteriovenous fistula or graft, AVF/AVG) patients initiating hemodialysis (HD) is superior to catheter access [1]. However, in spite of the 2003 Fistula First Initiative and subsequent programs, 80% patients initiating HD in the US continue to do so with catheters [2, 3].

Catheters are often thought the result of emergency HD initiation among patients with late or no pre-dialysis nephrology care, but this assumes time in nephrology care as the primary determinant for initiating HD with AVF/AVG. Administrative data from the US Centers for Medicare and Medicaid Services Form 2728 broadly captures if a patient was in the care of a nephrologist prior to starting dialysis, but lacks details on the role of patient engagement in that care and decision-making in provider or patient activation. The purpose of this retrospective chart review study was to examine to what extent patient engagement in pre-dialysis nephrology care and decision-making among patients who initiated HD with a catheter predicted provider and patient action towards obtaining AVF/AVG.

## Methods

### Study sample

We conducted a retrospective medical record review of adults (age 18–89 years) patients with CKD who received care in academically affiliated private practice, public hospital, or Veterans Administration settings prior to initiating HD between 10/1/2011 and 9/30/2012. After receiving standardized training in abstraction, 2 independent abstractors at each site conducted medical record reviews with post-abstraction adjudication using standard medical record abstraction forms. A total of 96 medical records were extracted. We excluded 4 records (3 public hospital, 1 Veterans Administration) for which date of dialysis initiation could not be confirmed. This study was approved at each site’s Institutional Review Board (Johns Hopkins University #00058006, University of California San Francisco #19–27,429, Nashville Veterans Administration Medical Center #691542). Each site’s Institutional Review Board approved a waiver of consent to access administrative records for this study. All methods were carried out in accordance with relevant guidelines and regulations.

### Primary outcomes

We examined two outcomes among patients who initiated HD with a catheter: (1) provider action towards AVF/AVG access creation; and (2) patient action toward AVF/AVG use prior to HD initiation. We defined provider action as documented referral for AVF/AVG creation prior to HD initiation. We defined patient action as documented completion of any step—i.e. evaluation by a vascular surgeon, vein mapping or vascular surgery—towards AVF/AVG creation.

### Primary predictors

Our primary predictors were (1) patient engagement in nephrology care within 6 months prior to HD initiation and (2) timing of patient modality decision-making. We defined patient engagement in care as a 4-level categorical variable reflecting the presence, consistency and recency of their interactions with pre-dialysis nephrology care: none (no pre-dialysis nephrology care); interrupted (span of care less than 3 months and last visit more than 30 days of HD initiation); limited (span of care less than 3 months but last visit within 30 days of HD initiation); and ideal (span of care more than 3 months and last visit within 30 days of HD initiation). We used these parameters because referral to nephrology care is considered late if within 3 months of HD initiation and a last visit prior to HD initiation more than 30 days suggests avoidance of care. We defined timing of patient dialysis modality decision was defined as a 4-level categorical variable reflecting the presence or recency of a decision reflecting patients’ treatment modality choices prior to initiating dialysis: no documentation of decision; late decision (less than 3 months before HD initiation); decision 3–12 months and more than 12 months of HD initiation.

### Covariates

Covariates were assessed via medical record review and included patients’ demographics (age, gender, and race), Charlson comorbidity index score and insurance status (private/Medicare/dual Medicare-Medicaid; Medicaid-only; military; uninsured). We also assessed the location of patients’ first dialysis (outpatient or inpatient), patients’ most recent laboratory measurement of kidney function prior to starting dialysis, documentation of unattended nephrology clinic visits, and clinician documentation of patients’ receipt of RRT options education.

### Statistical analysis

We restricted analyses to patients who initiated HD with a catheter. We described patient characteristics among those who initiated HD with a catheter were examined by clinical site. We used chi-square test for non-continuous variables and Kurskal-Wallis for continuous variables.

We performed multivariable logistic regression to examine the association of patient level of engagement in pre-dialysis nephrology care and timing of patient dialysis modality decision with provider or patient action. Models were minimally adjusted for patients’ age, insurance (any insurance vs no insurance), and Charlson comorbidity index score. Because dialysis and/or AVF/AVG may have not been considered consistent with the goals of care for patients over age 75 years, we conducted a subgroup analysis excluding this group of patients.

## Results

We examined the records of 92 incident HD patients with confirmed dialysis start dates between 10/1/2011 and 9/30/2012. Most patients received care in the private practice site (*n* = 39, 42%), while fewer received care in the public hospital site (*n* = 35, 38%) and Veterans Administration (*n* = 18, 20%) sites. Overall 66% (*n* = 61) of patients initiated HD with a catheter. The proportion of patients who initiated HD with a catheter was similar at the private practice (*n* = 28, 72%) and public hospital (*n* = 27, 77%) sites, but only a third of Veterans Administration patients (*n* = 6, 33%) did so. Estimated GFR at HD initiation was lower for those starting with a catheter [median 5 (IQR 3, 9)] than those starting with AVF/AVG [median 9 (IQR 5, 10)], *p* = 0.05. These data were not available for more than half of patients overall [*n* = 16 of 31 (52%) and 35 of 61 (57%) missing for patients with AVF/AVG and catheter, respectively) and primarily for patients in the private practice site (*n* = 38 of 39 missing, 97%). Estimated GFR at HD initiation data were unavailable for 12 of 35 patients at the public hospital site (34%) and 1 of 17 patients at the Veterans Administration site (6%).

**Fig. 1:**
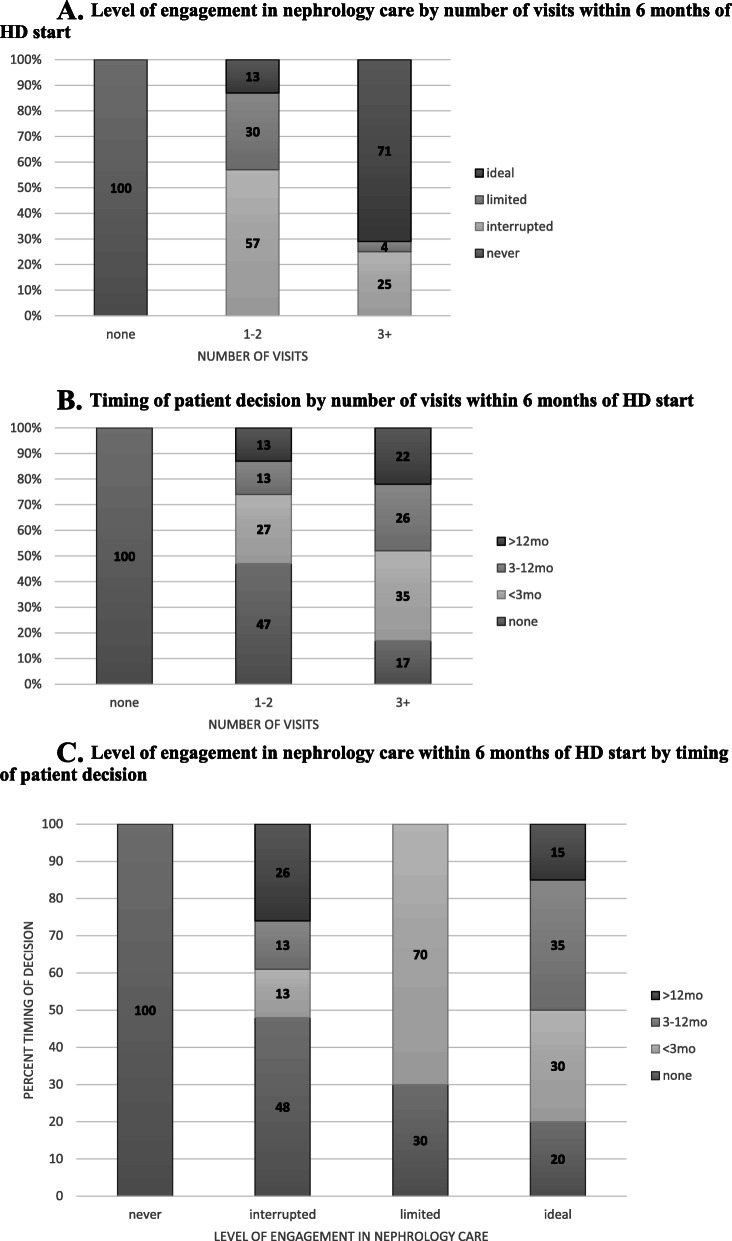
**a**. Level of engagement in nephrology care by number of visits within 6 months of HD start. **b**. Timing of patient decision by number of visits within 6 months of HD start. **c.** Level of engagement in nephrology care within 6 months of HD start by timing of patient decision

Characteristics of patients who initiated HD with a catheter are shown in Table 1. All Hispanic patients and all but one Asian patient received care at the public hospital site. All uninsured patients were at the public hospital. Level of patient engagement in care, timing of patient decision, and number of visits were similar across clinical sites. Twelve percent (*n* = 7) of patients had no pre-dialysis nephrology care, 38% (*n* = 23) had interrupted care, 16% (*n* = 10) had limited care, and 34% (*n* = 21) had ideal engagement in care. Roughly 81% (*n* = 17) of patients with ideal care and 26% (*n* = 6) of patients with interrupted care had more than 3 visits within 6 months of initiating HD (Fig. 1a). About 42% (*n* = 25) of patients had no documented decision, while 27% (*n* = 16), 17% (*n* = 10), and 15% (*n* = 9) had documentation of decision within 3 months, between 3 and 12 months, and more than 12 months of starting dialysis. Roughly one-fifth of patients with more than 3 visits within 6 months of initiating HD did not have a documented decision (Fig. 1b). Among patients with ideal level of engagement in care, 20% (*n* = 4) had no documented decision and 30% (*n* = 6) had documentation of decision within 3 months of initiating HD. (Fig. 1c).

**Table 1:** Catheter-start patient characteristics by clinical setting

Characteristic n (% ***column***)	Overall 61 (100)	Private practice 28 (46)	Public hospital 27 (44)	Veterans Administration 6 (10)	***p***-value
Level of engagement in nephrology care before HD start					0.3
None	7 (11)	2 (7)	4 (15)	1 (17)	
Interrupted (last > 30 days)	23 (38)	14 (50)	8 (30)	1 (17)	
Limited (<3mo, last within 30 days)	10 (16)	4 (14)	6 (22)	0 (0)	
Ideal (>3mo, last within 30 days)	21 (34)	8 (29)	9 (33)	4 (67)	
Timing of patient decision before HD start					0.5
No decision	25 (42)	11 (39)	11 (42)	3 (50)	
< 3 months	16 (27)	5 (18)	9 (35)	2 (33)	
3–12 months	10 (17)	7 (25)	2 (8)	1 (17)	
> 12 months	9 (15)	5 (18)	4 (15)	0 (0)	
Visits within 6 months of dialysis initiation					0.7
0	7 (11)	2 (7)	4 (15)	1 (17)	
1–2	30 (49)	16 (57)	11 (41)	3 (50)	
3+	24 (39)	10 (36)	12 (44)	2 (33)	
Age, mean (SD)	62 (15)	67 (14)	52 (13)	66 (10)	< 0.001
Male gender	33 (54)	11 (39)	16 (59)	6 (100)	0.02
Race/ethnicity					< 0.001
White	8 (13)	3 (11)	2 (7)	3 (50)	
Black	30 (49)	24 (86)	3 (11)	3 (50)	
Hispanic	11 (18)	0 (0)	11 (41)	0 (0)	
Asian	12 (20)	1 (4)	11 (41)	0 (0)	
eGFR at HD initiation, median (IQR)^a^	5.0 (3.0, 9.0)	n/a	4.5 (3, 7.5)	8.0 (5.0, 9.0)	0.1
Charlson comorbidity index score, median (IQR)	5.0 (3.0, 6.0)	6.0 (5.0, 8.0)	4.0 (2.0, 5.0)	4.5 (3.0, 6.0)	< 0.001
Uninsured	7 (11)	0 (0)	7 (26)	0 (0)	0.007
Inpatient start	30 (49)	5 (18)	19 (71)	6 (100)	< 0.001

*AVF/AVG* arteriovenous fistula/graft, *HD* hemodialysis, *RRT* renal replacement therapy, *eGFR* estimated glomerular filtration rate

^a^Data available: *n* = 26 overall, *n* = 0 private practice site, *n* = 20 public hospital site, *n* = 6 Veterans Administration

**Fig. 2:**
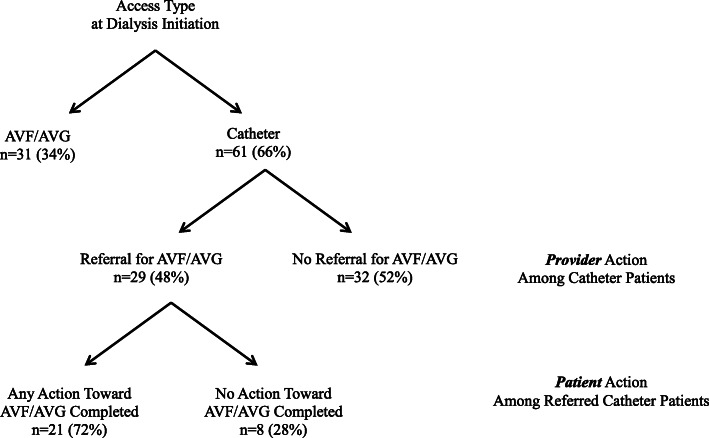
Provider and patient action toward AVF/AVG

Among patients initiating HD with a catheter, providers referred 48% (*n* = 29) of patients for AVF/AVG creation. Of those referred, 72% (*n* = 21) of patients took some action toward having an AVF/AVG created. (Fig. 2).

In unadjusted analysis, provider action toward AVF/AVG placement was not associated with interrupted (odds ratio (OR) [95% confidence interval (CI)]: 6.5 [0.7, 63.3], *p* = 0.1) or limited (OR [95% CI]: 2.6 [0.2, 31.7], *p* = 0.5) patient engagement in care, but it was statistically significantly associated with ideal engagement in care (OR [95% CI]: 9.8 [1.0, 96.6], *p* = 0.05) compared to patients with no pre-dialysis nephrology care. After adjustment, the association of provider action with ideal level of engagement in care strengthened (adjusted OR [95% CI]: 13.7 [1.08, 175.1] *p* = 0.04), but was not statistically significantly associated with other levels of engagement. In unadjusted analysis, patient action was not associated with level of engagement in care (interrupted OR [95% CI]: 1.6 [0.2, 10.1], *p* = 0.6; limited OR [95% CI]: 0.6 [0.06, 6.0], *p* = 0.7; ideal OR [95% CI]: 1.9 [0.3, 12.0], *p* = 0.5, compared to patients with no pre-dialysis nephrology care). The lack of association of patient action with level of engagement of care persisted in adjusted models.

**Fig. 3:**
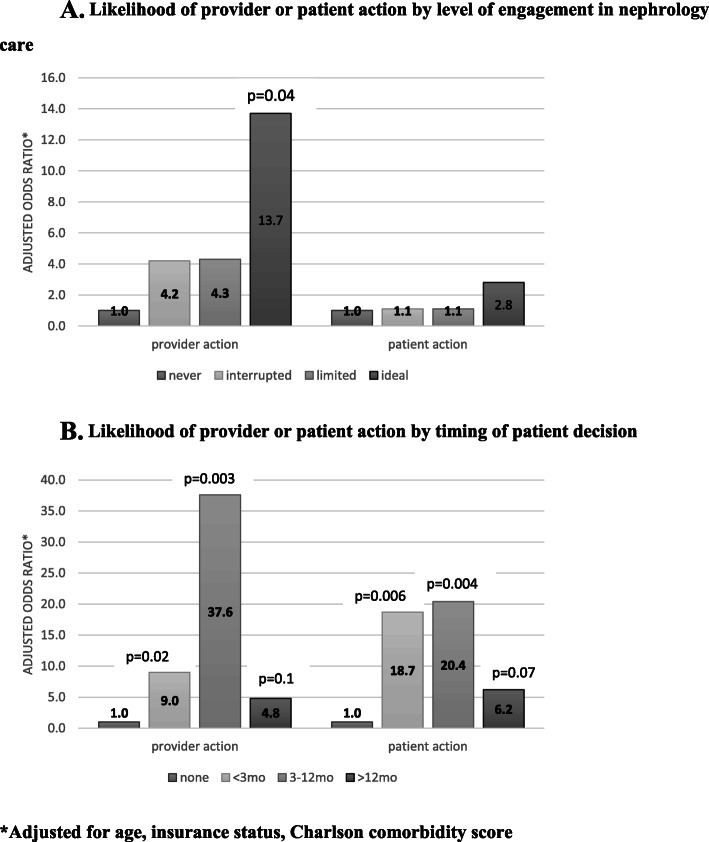
**a**. Likelihood of provider or patient action by level of engagement in nephrology care. **b**. Likelihood of provider or patient action by timing of patient decision. *Adjusted for age, insurance status, Charlson comorbidity score

Compared to patients with no documented decision, patients who had documented decisions within 3 months, 3–12 months, or more than 12 months before initiating dialysis were more likely to have provider action toward AVF/AVG (OR [95% CI]: 4.1 [1.1, 15.7], *p* = 0.04; OR [95% CI]: 28.5 [3.0, 273.3], *p* = 0.004), and OR [95% CI]: 4.0 [0.8, 19.7], *p* = 0.9, respectively). After adjustment, provider action toward AVF/AVG was more likely among patients who had a documented decision within 3 months, 3–12 months, or more than 12 months before starting HD when compared to patients with no documented decision (adjusted OR [95% CI]: 9.0 [1.4, 55.6], *p* = 0.2), 37.6 [3.3, 423.4], *p* = 0.003), and 4.8 [0.8, 30.6], *p* = 0.1, respectively). Similarly, patient action toward AVF/AVG was more likely with a documented decision. In unadjusted analysis, patient action toward AVF/AVG was more likely among patients who had a documented decision within 3 months, 3–12 months, or more than 12 months before starting HD when compared to patients with no documented decision (adjusted OR [95% CI]: 4.1 [1.0, 17.5], *p* = 0.6, 12.2 [2.2, 68.7], *p* = 0.004, and 4.2[0.8, 22.9], *p* = 0.1, respectively). After adjustment, patient action toward AVF/AVG was more likely among patients who had a documented decision within 3 months, 3–12 months, or more than 12 months before starting HD when compared to patients with no documented decision (adjusted OR [95% CI]: 18.7 [2.3, 149.0], *p* = 0.006, 20.4 [2.6, 160.0] *p* = 0.004), and 6.2 [0.9, 44.0], *p* = 0.07, respectively). (Fig. 3a and b).

Among all patients, there were only 15 patients over age 75 years, most of whom (*n* = 11, 73%) initiated HD with a catheter. In a subgroup analysis excluding those over age 75 years (*n* = 50), findings did not change appreciably.

## Discussion

Ideally, most patients initiating HD should do so in a planned fashion and with a functioning AVF/AVG to minimize morbidity and mortality [4, 5]. This requires significant planning and, depending on individual practice, resources including surgeon availability and nephrology care prior to HD initiation [6, 7]. Prior literature has focused primarily on the duration of pre-dialysis nephrology care and its association with patients initiating HD with AVF/AVG [8–11]. In this retrospective medical record review study of a diverse cohort of incident HD patients from between 2011 and 2012, we delved further through investigation of the level of engagement in pre-dialysis nephrology care and timing of patient modality decision-making. We found that 66% started dialysis with a catheter, which was lower than the national standard of 80% [12]. However, among those initiating with a catheter, only 48% of providers referred patients for AVF/AVG. Nearly two-thirds of referred patients took at least one action (e.g. attending surgical appointment, having vein mapping, or having vascular surgery) toward receiving AVF/AVG.

Our study demonstrates that access to nephrology care at least 6 months prior to HD start is insufficient. We found that only a third of patients were engaged in an ideal level of care (at least 3 months and within 30 days of HD start), while roughly half had either no pre-dialysis nephrology care or pre-dialysis nephrology care that was interrupted. Patients with interrupted patterns of care may have left clinic with no intention of returning or faced challenges scheduling return appointments. Individuals who are referred but do not attend or return to nephrology care may be afraid of the prospect of impending dialysis-requiring kidney failure and/or may have more competing basic life issues common to the poor and ethnically diverse communities [13]. Efforts to contact patients lost to follow-up and address barriers to returning to care are needed and could be facilitated by system level interventions such as surveillance registries designed to identify individuals eligible for AVG/AVF [14].

Ideally providers should have referred all patients starting HD for AVF/AVG. The process of referral presents an opportunity for providers to discuss the importance of avoiding a dialysis catheter and self-care behaviors to preserve the vascular network, thus improving the chances for successful AVF maturation [15]. Suboptimal referrals may be due a lack of coordinated care systems, a lack of access to timely nephrology care, delayed patient referrals from primary care to nephrology care, and rapid and unexpected kidney function decline. Patient indecision regarding preferred RRT or refusal of referral appears to be an important factor given the association with provider action. Not surprisingly, timing of decision was also associated with patient action, suggesting patients who have made a modality decision may be more psychologically prepared to pursue physical action. But given that nearly half of patients with interrupted care had no documented decision and half of patients with ideal level of engagement of care either had no documented decision or a late decision suggests additional efforts to motivate patients to make decisions are needed [16]. Studies suggest patients’ lack of symptoms until very late in the course of end-stage kidney disease could lead patients to ignore or deny the gravity of their illness [17]. Efforts to heighten general awareness of the need to seek nephrology care and motivational interviewing, even in the absence of symptoms, could help to address these issues.

Our study cohort was drawn from three distinct patient populations and locales, allowing us to achieve high demographic diversity as well as diversity in clinical practice settings, thus making our findings more generalizable. However, this study is not without limitations. First, the cohort is relatively small, which may have limited our power to detect an association between nephrology visit intensity and patients taking any action toward AVF/AVG placement, if one existed. Second, our study was conducted several years ago, but guidelines for RRT preparation have not changed in the intervening time and timely creation of AVF/AVG remains suboptimal among patients with ESKD [2]. Third, we lacked data to determine the trajectory for kidney function decline. The prognostic information provided by the eGFR trajectory may help clarify optimal timing for AVF/AVG referral and improve patients’ understanding for the need of vascular access planning [18]. However, our finding that two-thirds of patients initiating HD with a catheter had no, interrupted, or limited engagement in pre-dialysis nephrology care, suggests efforts to get and retain patients in care are needed. Finally, we did not collect data on availability of vascular surgery services, trajectory of kidney function decline, or regarding the reasons underlying providers’ or patients’ actions or lack thereof. The latter would be more conducive to a qualitative study design.

In conclusion, rates of referral for AVF/AVG creation were suboptimal among nephrologists for patients who initiated HD via catheter, but both provider and patient action were more likely when patients had a documented decision about their upcoming treatment modality. Research into how to best encourage patients to engage in and stay in nephrology care to facilitate earlier treatment decisions may, therefore, benefit efforts to improve patients’ rates of planned HD initiation with permanent vascular access.

## Data Availability

The datasets used and/or analysed during the current study are available from the corresponding author on reasonable request.
